# Design, synthesis, and biological evaluation of novel urolithins derivatives as potential phosphodiesterase II inhibitors

**DOI:** 10.1038/s41598-021-03194-y

**Published:** 2021-12-10

**Authors:** Long Tang, Jianchun Jiang, Guoqiang Song, Yajing Wang, Ziheng Zhuang, Ying Tan, Yan Xia, Xianfeng Huang, Xiaoqing Feng

**Affiliations:** 1 Institute of Chemical Industry of Forest Products, Chinese Academy of Forestry, Nanjing, 210042 China; 2 School of Chemical Engineering, Nanjing Forestry University, Nanjing, 210037 China; 3 School of Pharmaceutical Engineering & Life Science, Changzhou University, Changzhou, 213164 China

**Keywords:** Drug discovery, Neuroscience

## Abstract

A series of urolithins derivatives were designed and synthesized, and their structures have been confirmed by ^1^H NMR, ^13^C NMR, and HR-MS. The inhibitory activity of these derivatives on phosphodiesterase II (PDE2) was thoroughly studied with 3-hydroxy-8-methyl-6*H*-benzo[C]chromen-6-one and 3-hydroxy-7,8,9,10-tetrahydro-6*H*-benzo[C] chromen-6-one as the lead compounds. The biological activity test showed that compound **2e** had the best inhibitory activity on PDE2 with an IC_50_ of 33.95 μM. This study provides a foundation for further structural modification and transformation of urolithins to obtain PDE2 inhibitor small molecules with better inhibitory activity.

## Introduction

Ellagic acid (EA) is a polyphenol dilactone-based natural active substance which owns several pharmacological characteristics of antioxidant, anti-tumor, anti-mutation, antibacterial, anti-inflammatory effects, and so on^1–6^. Recently, it was been reported that EA can reduce the formation of advanced glycation end products inducing autophagy cells which protect the hippocampus of brain, suggesting great therapeutic potential in the treatment of progressive neurodegenerative diseases such as typical Alzheimer's disease (AD) and Parkinson's disease (PD)^7^. However, there still remains deficiencies that limit the application of EA in the pharmaceutical field, specifically, poor solubility and low bioavailability^1,7^.

**Figure 1:**
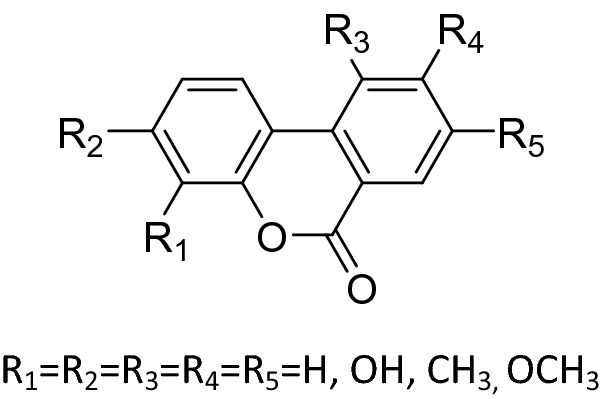
Chemical structure of urolithins compounds.

Urolithins are bioavailable, which are intestinal metabolites of EA^8–10^. Urolithins have various biological activities including cardiovascular protection, anti-inflammatory activity, anticancer properties, antidiabetic activity, antiaging properties and et al.^11,12^. Urolithins have become bioactive markers of EA exposure^2,10–14^, and since it is bioavailable, it also shows great potential in the treatment of progressive neurodegenerative diseases^15–21^. Dozens of urolithins structures have been discovered^22–24^; such as Urolithin A, Urolithin B and Methylurolithin (Fig. 1).

AD is a degenerative disease of the central nervous system whose incidence continues to rise. AD seriously affects the normal life of patients, especially that of the elderly in later years. Despite its prevalence and severity, there is no proven method to delay its onset or progress^25–28^. Although the drug GV-971 (sodium oligomannate capsules), developed by Green Valley Pharmaceutical, and was approved by the NMPA for conditional marketing in November 2019, there remains doubts on the effectiveness of its treatment. The lack of proven treating methods continues to be one of the biggest challenges for the effective intervention and reversal of the increasing occurrence and development of AD today.

A potential drug target, phosphodiesterase II (PDE2), is mainly distributed in brain and heart cells. PDE2 maintains the levels of intracellular second messenger cyclic adenosine monophosphate (cAMP) and cyclic guanosine monophosphate (cGMP) through catalyzing the hydrolysis of cAMP and cGMP^2,29–31^. It has been commonly recognised in pharmaceutical industry that selective inhibitors of PDE2 are of great significance and value in improving the permeability and memory of endothelial cells, thus intervening and even reversing the progress of AD. Discovery of the composite crystal structure of PDE2 and the highly selective inhibitor BAY60-7550 (protein number: 4HTX) provided a basis for rational design of small-molecule ligands targeted to PDE2 in 2013^32^. Unfortunately, there is no drug successfully marketed so far.

In this study, a series of derivatives of the lead compounds, i.e. 3-hydroxy-8-methyl-6*H*-benzo[C]chromen-6-one (IC_50_ > 100 μM) and 3-hydroxy-7,8,9,10-tetrahydro-6*H*-benzo[C]chromen-6-one (IC_50_ = 93.24 μM) were designed based on the idea of the five-point rule of drug design and preliminary research^31^. The designed compounds were employed to combine with the PDE2 protein crystal structure 4HTX in Discovery Studio software to screen out which one bears higher affinity to the specific receptor binding region of 4HTX. Finally, the compounds were futher synthesized and their activities were verified at the enzyme level to determine which one had better PDE2 inhibitor activity.

## Results

### Synthetic pathways

**Scheme 1:**
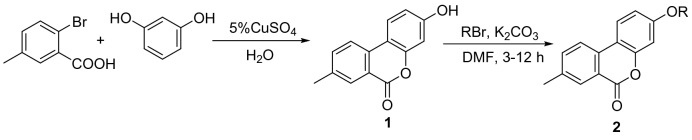
Synthesis of 3-hydroxy-8-methyl-6H-benzo[c]chromen-6-one derivatives. Reagents and conditions: (**1**) 5% CuSO_4_, NaOH, H_2_O, reflux, 15 h; (**2**) K_2_CO_3_, DMF, 120 °C, 3–12 h.

**Scheme 2:**
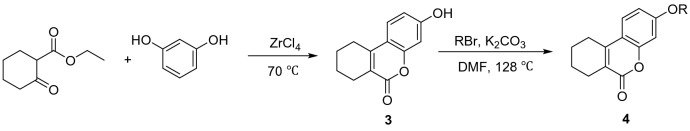
Synthesis of 3-hydroxy-7,8,9,10-tetrahydro-6H-benzo[c]chromen-6-one derivatives. Reagents and conditions: (**3**) ZrCl_4_, 70 °C, 1–2 h; (**4**) K_2_CO_3_, DMF, 128 °C, 3–6 h.

Our purpose was to introducing different substituents into the hydroxy scaffold of urolithins at the 3-position while keeping the lactone ring still. As such, various novel 3-hydroxy-8-methyl-6*H*-benzo[c]chromen-6-one and 3-hydroxy-7,8,9,10-tetrahydro-6*H*-benzo[c] chromen-6-one derivatives were designed and synthesized. The general pathways for synthesizing 3-hydroxy-8-methyl-6H-benzo[c]chromen-6-one derivatives **2** and 3-hydroxy-7,8,9,10-tetrahydro-6*H*-benzo[c]chromen-6-one derivatives **4** are shown in Schemes 1^33^ and 2^34^, respectively. In Scheme 1, the reaction of 2-bromobenzoic acid and resorcinol in the presence of CuSO_4_ and NaOH produces intermediate **1**. In Scheme 2, the reaction of ethyl 2-oxocyclohexanecarboxylate and resorcinol in the presence of ZrCl produces intermediate **3**. Intermediate **1** and **3** were then reacted with various halides including alkyl bromides and heterocyclic bromides to generate the desired target products (3-hydroxy-8-methyl-6*H*-benzo[c]chromen-6-one derivatives **2** and 3-hydroxy-7,8,9,10-tetrahydro-6*H*-benzo[c]chromen-6-one derivatives **4**) in moderate to good yields.

### Discovery studio CDOCKER program molecular docking results

In order to verify the binding effect of the designed derivatives with the PDE2 protein crystal structure 4HTX, and to screen out any derivatives with high affinity for the specific receptor binding region of 4HTX, the CDOCKER module of the computer-aided design software Discovery Studio was used to compare the PDE2 protein crystal structure 4HTX with the structure of the derivatives. All of the docking results are presented as -CDOCKER_INTERACTION_ENERGY; the larger values of -CDOCKER_INTERACTION_ENERGY represent better affinity of the derivative for 4HTX. Before docking our main compounds, we have verified the affinity of 4HTX protein and the small molecule BAY60-7550 in its crystal structure, and the -CDOCKER_INTERACTION_ENERGY value is 57.95. TThe designed etherification groups are mainly C2–C9 alkyls, cycloalkyl-substituted C1–C9 alkyls, heterocyclyl-substituted C1–C9 alkyls, and aryl-substituted C1–C9 alkyls or C2–C9 hetero-ring bases. The 3-hydroxy-8-methyl-6*H*-benzo[C]chromen-6-one derivatives and 3-hydroxy-7,8,9,10-tetrahydro-6*H*-benzene[C]chromen-6-one derivatives received higher docking scores when etherification groups were C5–C7 substituted cycloalkyls, heterocyclic substitutions, and alkyls (Table 1). Possibly because the substituents composed of 5 to 7 carbons occupy pockets of the protein crystals. Moreover, the docking scores of the designed derivatives were higher than those of the lead compounds, indicating that the affinities of the designed derivatives to the protein crystal 4HTX are better than those of the lead compounds at the molecular docking level, providing theoretical support for the synthesis of the derivatives.

**Table 1:** Docking results of the compounds and 4HTX.

Compound	- CDOCKER_INTERACTION_ENERGY	Compound	- CDOCKER_INTERACTION_ENERGY
**1**	33.63	**3**	27.21
**2a**	30.26	**4a**	30.73
**2b**	33.56	**4b**	35.58
**2c**	34.65	**4c**	31.40
**2d**	33.05	**4d**	32.04
**2e**	38.46	**4e**	36.73
**2f**	36.57	**4f**	35.55
**2g**	38.46	**4g**	40.13
**2h**	38.74	**4h**	39.39
**2i**	40.75	**4i**	36.92
**2j**	39.69	**4j**	39.68
**2k**	39.74	**4k**	38.31
**2l**	37.06	**4l**	37.36
**2m**	44.50	**4m**	42.98
**2n**	43.16		

### Enzymatic assays

**Figure 2:**
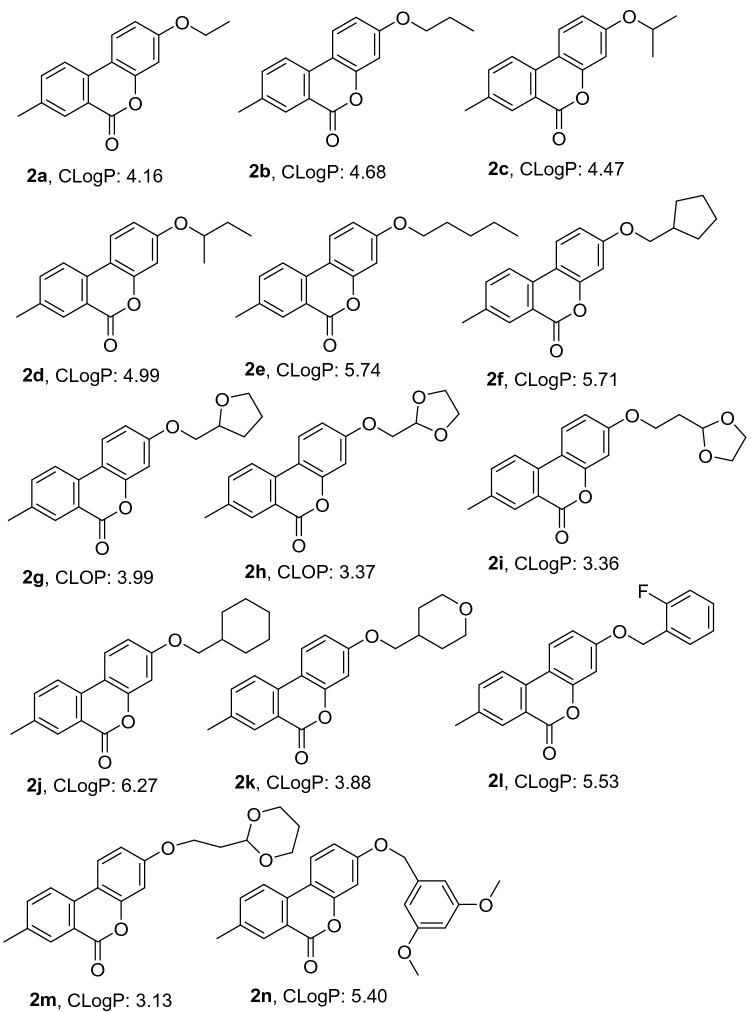
CLoP values of 3-hydroxy-8-methyl-6H-benzo[C]chromen-6-one derivatives **2.**

**Figure 3:**
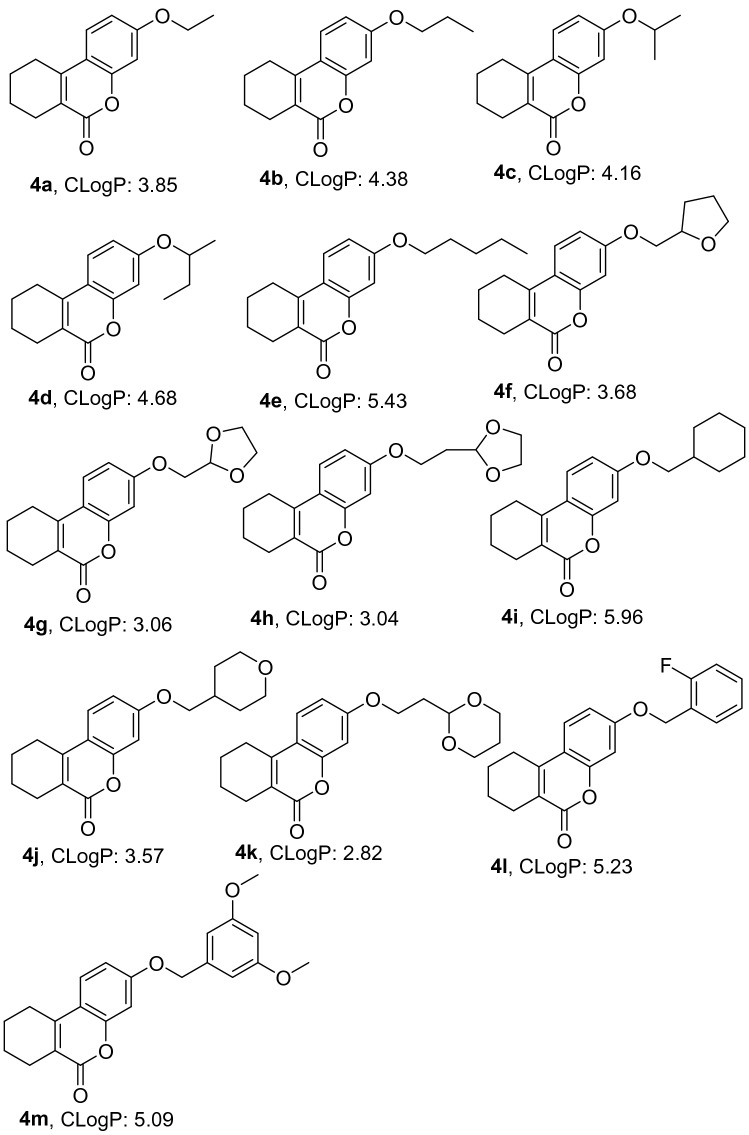
CLoP values of 3-hydroxy-7,8,9,10-tetrahydro-6*H*-benzo[C]chromen-6-one derivatives **4.**

Figures 2 and 3 illustrates that most of the derivatives produce appropriate values of ClogP (2.0–5.0), indicating good blood–brain barrier penetration. The AlphaScreen kit method was used to detect PDE2 inhibitory activity of the 27 synthesized compounds **(2a–n** and **4a–m**) in vitro, each diluted at seven concentrations, and each carried out with three sets of parallel experiments. The IC_50_ values were obtained by fitting calculations using software Graphpad Prism 7. The results showed that R group composition remarkably affects the PDE2 inhibitory activities (Table 2). When the R groups contains less than five carbons, the PDE2 inhibitory activity was relatively good; compounds **2e** and **4c** affect the PDE2 inhibitory activities significantly with IC_50_ values of 33.95 μM and 34.35 μM, respectively. Despite the good PDE2 inhibitory activity in compounds **2e** and **4c**, these compounds must be modified furtherly.

**Table 2:** In vitro PDE2 inhibitory activity of 6*H*-benzo[*c*]chromen-6-one derivatives **2** and **4**.

Compound	PDE2 IC_50_^a^ (μM)	Compound	PDE2 IC_50_^a,b^ (μM)
**1**	> 100	**3**	93.24
**2a**	> 100	**4a**	> 100
**2b**	> 100	**4b**	> 100
**2c**	> 100	**4c**	34.35
**2d**	> 100	**4d**	> 100
**2e**	**33.95**	**4e**	> 100
**2f**	> 100	**4f**	> 100
**2g**	> 100	**4g**	> 100
**2h**	> 100	**4h**	> 100
**2i**	> 100	**4i**	> 100
**2j**	**65.71**	**4j**	> 100
**2k**	> 100	**4k**	> 100
**2l**	> 100	**4l**	> 100
**2m**	> 100	**4m**	> 100
**2n**	> 100		

^a^BAY60-7550 was used as the reference compound with an IC_50_ of 8.4 nM.

^b^Results are expressed as the mean of at least three experiments.

Significant values are in bold.

The PDE2 inhibitory activity of the lead compounds 3-hydroxy-8-methyl-6*H*-benzo[C]chromen-6-one and 3-hydroxy-7,8,9,10-tetrahydro-6*H*-benzo[C] chromen-6-one were also tested at the enzyme level, IC_50_ values greater than 100 μM and 93.24 μM, respectively. It can be seen that the lead compounds have poor inhibitory activity on PDE2. To target find derivatives with better activity, a rational structure design on the lead compounds based on the basic idea of the five-point rule of drug design and the basis of preliminary research was carried out, and with the help of the CDOCKER module of the Discovery Studio professional software. 27 derivatives (Figs. 2 and 3) were synthesized and the inhibitory activity of on PDE2 at the enzyme level were verified. The **2e** and **4c** showed certain inhibitory effect on PDE2, with IC_50_ values of 33.95 μM and 34.35 μM, respectively. The inhibitory effect of other derivatives on PDE2 was not remarkable, which agrees with the results of molecular docking (See Table 1); that is, higher affinity of compound with 4HTX yields higher molecular docking score, smaller IC_50_ value, and better PDE2 inhibitory activity.

## Methods

### Synthesis

All chemicals, reagents, and solvents were analytical grade, purchased from commercial suppliers (i.e., Alfa, Meyer, and Aladdin).

^1^H NMR spectra were recorded on a Bruker DRX spectrometer at 300 and 400 MHz; ^13^C NMR spectra were recorded on a BrukerBioSpin GmbH spectrometer at 75 and 100 MHz; the coupling constants are given by Hz; The melting points were measured with an X4-A microscopic apparatus; Mass (HRMS) analysis was conducted by Agilent 6200 Accurate-Mass TOF LC/MS system with Electrospray Ionization (ESI); IR peaks appear were recorded on Fourier transformer infrared (FTIR) spectrometer (Thermo fisher, Nicolet iS50); The reactions were monitored via thin-layer chromatography (TLC) performed on GF254 plates and visualised under UV light^33^.

### Molecular docking program of discovery studio

**Figure 4:**
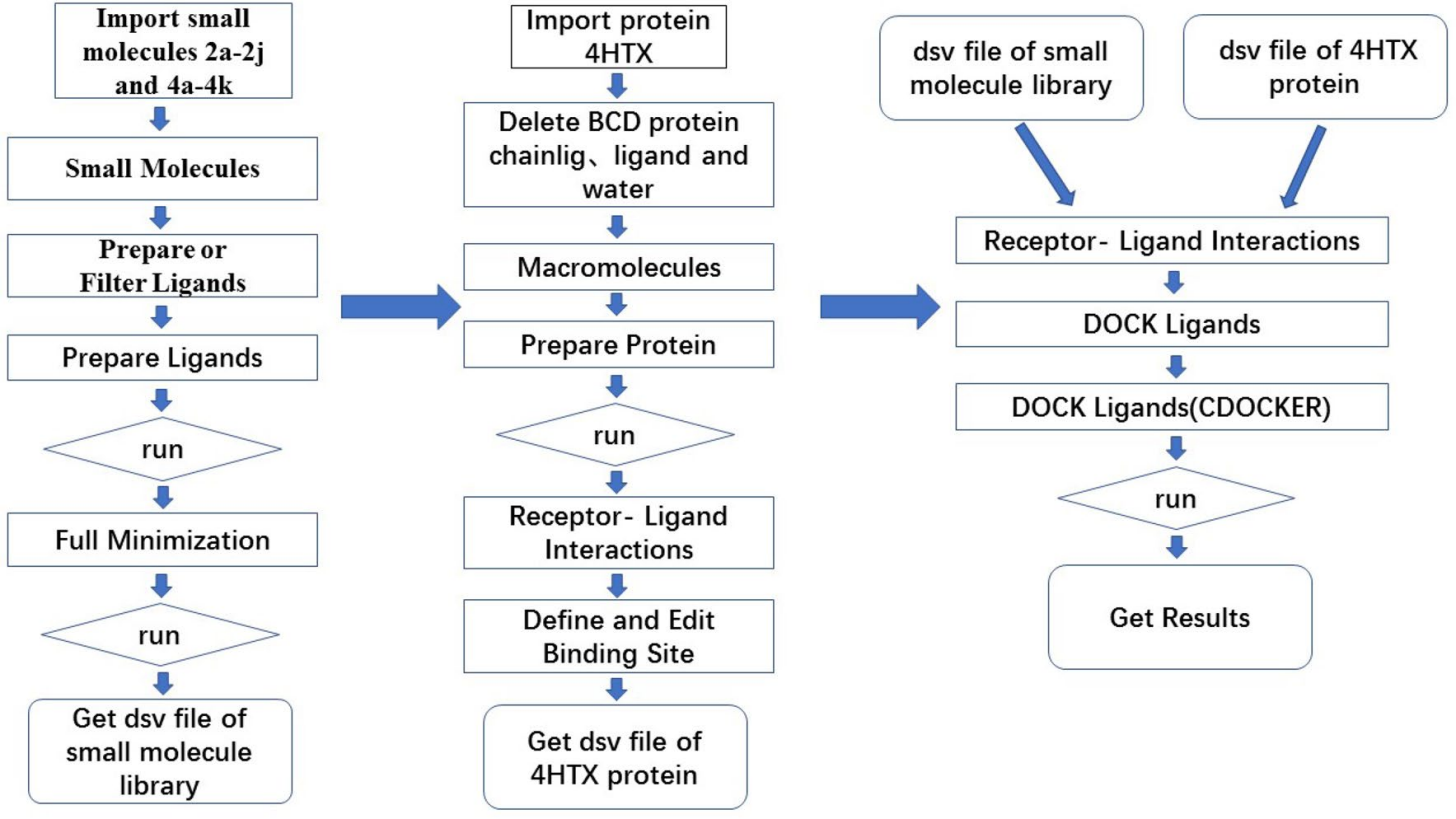
Molecular docking program of discovery studio.

Discovery Studio (version number:2020; URL: https://www.neotrident.com/product/proinfo/27.html) is a professional molecular simulation software which can locate the ligand molecule on the active site of a receptor and evaluate real-time interaction between ligand and the receptor. After several turns of screenings, the best binding mode between ligand and receptor can be found. The CDOCKER semi-flexible module was used in Discovery Studio to locate the designed series of derivatives on the active site of the PDE2 protein crystal structure 4HTX to screen the ligand small molecule derivatives and to get the -CDOCKER_INTERACTION_ENERGY function values. The specific process used is shown in Fig. 4.

### Synthetic method

#### Synthesis of 3-hydroxy-8-methyl-6*H*-benzo [C]chromen-6-one (1)

5-methyl-2-bromobenzoic acid (21.5 g, 100 mmol), resorcinol (22.5 g, 200 mmol), sodium hydroxide (8.25 g, 200 mmol), and water 250–380 mL were added to the flask. The solution was heated to 100 °C, stirred and refluxed for 20 min, and then 45 mL of 5% copper sulfate solution was added. The temperature was kept at 100 °C and the mixture was stirred for 15 h. The mixture was monitored by TLC. Upon reaction, the mixture was cooled to room temperature, filtered with suction, and the filter cake was washed with plenty of ice water until the filtrate was clear and transparent. A brown solid was obtained after air-drying at 40 °C. Methanol and acetic acid were then added for 1 h to recrystallise and to obtain a pink solid of 3-hydroxy-8-methyl-6H-benzo[C]chromen-6-one (6.52 g, 28.9%).

6-M. p. 266.8–267.5 °C; ^1^H NMR (300 MHz, DMSO-*d*_*6*_) *δ* 10.27 (s, 1H), 8.13 (dd, *J* = 9.0, 6.0 Hz, 2H), 7.98 (s, 1H), 7.70 (dd, *J* = 9.0, 3.0 Hz, 1H), 6.83 (dd, *J* = 9.0, 3.0 Hz, 1H), 6.74 (d, *J* = 2.4 Hz, 1H), 2.44 (s, 3H). ^13^C NMR (75 MHz, DMSO-*d*_*6*_) *δ* 165.87, 164.68, 157.02, 142.60, 141.62, 137.86, 134.50, 129.77, 126.90, 124.05, 118.30, 114.72, 108.11, 25.87.

#### Synthesis of 3-hydroxy-8-methyl-6*H*-benzo[C]chromen-6-one derivatives (2a–2n)

Hydroxy-8-methyl-6*H*-benzo[C]chromen-6-one (**1**; 2.0 g, 8.85 mmol), anhydrous potassium carbonate (1.6 g, 11.5 mmol), anhydrous DMF (100 mL), and bromide (11.5 mmol) were added into a reaction flask. The reaction mixture was heated to 120 °C and then stirred and refluxed for 3–12 h, monitored by TLC. The mixture was then poured into ice water, filtered with suction, and the filter cake was washed with water until the filtrate was clear and transparent. The filter cake was separated by column chromatography (PE:EA = 30:1) to obtain pure compounds.

3-ethoxy-8-methyl-6*H*-benzo[c]chromen-6-one (**2a**): Using bromoethane as the starting martial, the desired product **2a** was obtained as a white solid (1.0 g, 44.5%). M. p. 132.8–134.0 °C; IR characteristic peaks appear at (ν cm^−1^): 1736 (–C=O), 1169 (C–O–C), 1272, 1042 (Ar–O–R). ^1^H NMR (300 MHz, DMSO-*d*_*6*_) *δ* 8.23 (d, *J* = 3.0 Hz, 1H), 8.19 (d, *J* = 3.0 Hz, 1H), 8.01 (*s*, 1H), 7.73 (dd, *J* = 6.0, 2.1 Hz, 1H), 7.01–6.94 (m, 2H), 4.13 (dd, *J* = 15.0, 15.0 Hz, 2H), 2.45 (s, 3H), 1.37 (t, *J* = 7.0 Hz, 3H). ^13^C NMR (75 MHz, DMSO-*d*_6_) *δ* 161.02, 160.60, 152.21, 138.29, 136.88, 132.77, 129.76, 124.90, 122.41, 119.54, 113.01, 111.11, 102.28, 64.24, 21.15, 14.95; HRMS (ESI) *m/z* calcd for C_16_H_15_O_3_^+^[M + H]^+^: 255.0943 found: 255.0740.

8-methyl-3-propoxy-6H-benzo[c]chromen-6-one (**2b**): Using 1-bromopropane as the starting martial, the desired product **2b** was obtained as a white solid (1.33 g, 59.3%). M. p. 112.3–112.6 °C; IR characteristic peaks appear at (ν cm^−1^): 1726 (–C=O), 1172 (C–O–C), 1275, 1026 (Ar–O–R). ^1^H NMR (300 MHz, DMSO-*d*_*6*_) *δ* 8.21 (d, *J* = 3.0 Hz, 1H), 8.18 (d, *J* = 3.0 Hz, 1H), 8.00 (*s*, 1H), 7.72 (dd, *J* = 9.0, 3.0 Hz, 1H), 6.99–6.95 (m, 2H), 4.03 (t, *J* = 6.0 Hz, 2H), 2.45 (s, 3H), 1.82–1.71 (m, 2H), 1.00 (t, *J* = 7.5 Hz, 3H). ^13^C NMR (75 MHz, DMSO-*d*_6_) *δ* 161.03, 160.76, 152.22, 138.30, 136.90, 132.78, 129.77, 124.91, 122.43, 119.55, 113.06, 111.12, 102.33, 70.02, 22.36, 21.05, 10.82; HRMS (ESI) *m/z* calcd for C_17_H_17_O_3_^+^[M + H]^+^: 268.1099 found: 268.1095.

3-isopropoxy-8-methyl-6*H*-benzo[c]chromen-6-one (**2c**): Using 2-bromopropane as the starting martial, the desired product **2c** was obtained as a white solid (0.99 g, 42.1%). M. p. 102.3–103.0 °C; IR characteristic peaks appear at (ν cm^−1^): 1721 (–C=O), 1186 (C–O–C), 1267, 1015 (Ar–O–R). ^1^H NMR (300 MHz, DMSO-*d*_*6*_) *δ* 8.23 (d, *J* = 3.0 Hz, 1H), 8.19 (d, *J* = 3.0 Hz, 1H), 8.02 (s, 1H), 7.74 (dd,* J* = 6.0, 2.1 Hz, 1H), 7.07–6.88 (m, 2H), 4.77–4.73 (m, 1H), 2.46 (s, 3H), 1.31 (d, *J* = 6.0 Hz, 6H). ^13^C NMR (75 MHz, DMSO-*d*_*6*_) *δ* 161.06, 159.58, 152.30, 138.28, 136.91, 132.80, 129.77, 124.99, 122.42, 119.55, 113.83, 111.00, 103.28, 70.40, 22.15, 21.15; HRMS (ESI) *m/z* calcd for C_17_H_17_O_3_^+^[M + H]^+^: 268.1099 found: 268.1095.

3-(sec-butoxy)-8-methyl-6*H*-benzo[c]chromen-6-one (**2d**): Using 2-bromobutane as the starting martial, the desired product **2d** was obtained as a white solid (1.28 g, 51.7%). M. p. 62.6–63.4 °C; IR characteristic peaks appear at (ν cm^−1^): 1719 (–C=O), 1176 (C–O–C), 1269, 1018 (Ar–O–R). ^1^H NMR (300 MHz, DMSO-*d*_*6*_) *δ* 8.22 (d, *J* = 3.0 Hz, 1H), 8.19 (d, *J* = 6.0 Hz, 1H), 8.02 (s, 1H), 7.74 (dd, *J* = 6.0, 2.1 Hz, 1H), 6.99–6.96 (m, 2H), 4.58–4.54 (m, 1H), 2.46 (s, 3H), 1.72–1.59 (m, 2H), 1.28 (d, *J* = 3.0 Hz, 3H), 0.95 (t, *J* = 6.0 Hz, 3H). ^13^C NMR (75 MHz, DMSO-*d*_*6*_) *δ* 161.06, 159.92, 152.30, 138.26, 136.90, 132.80, 129.77, 125.00, 122.41, 119.55, 113.84, 111.01, 103.29, 75.23, 28.91, 21.15, 19.39, 9.94; HRMS (ESI) *m/z* calcd for C_18_H_19_O_3_^+^[M + H]^+^: 282.1256found: 282.1250.

8-methyl-3-(pentyloxy)-6*H*-benzo[c]chromen-6-one (**2e**): Using 1-bromopentane as the starting martial, the desired product **2e** was obtained as a white solid (0.92 g, 35.3%). M. p. 90.3–91.6 °C; IR characteristic peaks appear at (ν cm^−1^): 1725 (–C=O), 1172 (C–O–C), 1265, 1028 (Ar–O–R). ^1^H NMR (300 MHz, DMSO-*d*_*6*_) *δ* 8.23 (d, *J* = 3.0 Hz, 1H), 8.21 (d, *J* = 6.0 Hz, 1H), 8.02 (s, 1H), 7.74 (dd, *J* = 6.0, 2.1 Hz, 1H), 6.99–6.97 (m, 2H), 4.07 (t, *J* = 4.5 Hz, 2H), 2.46 (s, 3H), 1.79–1.72 (m, 2H), 1.46–1.32 (m, 4H), 0.91 (t, *J* = 6.0 Hz, 3H). ^13^C NMR (75 MHz, DMSO-*d*_6_) *δ* 161.04, 160.78, 152.24, 138.31, 136.92, 132.80, 129.78, 124.92, 122.45, 119.57, 113.08, 111.13, 102.34, 68.57, 28.69, 28.13, 22.35, 21.15, 14.39; HRMS (ESI) *m/z* calcd for C_19_H_21_O_3_^+^[M + H]^+^: 296.1412 found: 296.1409.

3-(cyclopentylmethoxy)-8-methyl-6*H*-benzo[c]chromen-6-one (**2f**): Using (bromomethyl)cyclopentane as the starting martial, the desired product **2f** was obtained as a white solid (1.39 g, 50.6%). M. p. 122.8–123.6 °C; IR characteristic peaks appear at (ν cm^−1^): 1725 (–C=O), 1171 (C–O–C), 1264, 1031 (Ar–O–R). ^1^H NMR (300 MHz, DMSO-*d*_*6*_) *δ* 8.22 (dd, *J* = 9.0, 3.0 Hz, 2H), 8.02 (s, 1H), 7.73(dd, *J* = 9.0, 3.0 Hz, 1H), 6.99–6.96 (m, 2H), 3.95(d, *J* = 9.0, 3.0 Hz, 2H), 2.45 (s, 3H), 2.38–2.28 (m, 1H), 1.83–1.74 (m, 2H),1.64–1.52 (m, 4H), 1.41–1.30 (m, 2H); ^13^C NMR (75 MHz, DMSO-*d*_*6*_) *δ* 161.02, 160.64, 152.17, 138.36, 136.91, 132.74, 129.77, 124.92, 122.46, 119.59, 113.10, 111.30, 102.45, 76.80, 71.11, 67.98, 28.06, 25.67, 21.15; HRMS (ESI) m/z calcd for C_19_H_19_O_4_^+^[M + H]^+^: 311.1205 found: 311.1201.

8-methyl-3-((tetrahydrofuran-2-yl) methoxy)-6*H*-benzo[c]chromen-6-one (**2g**): Using 2-(bromomethyl)-tetrahydrofuran as the starting martial, the desired product **2g** was obtained as a white solid (1.35 g, 49.4%). M. p. 122.8–123.6 °C; IR characteristic peaks appear at (ν cm^−1^): 1725 (–C=O), 1171 (C–O–C), 1267, 1042 (Ar–O–R). ^1^H NMR (300 MHz, DMSO-*d*_*6*_) *δ* 8.22 (dd, *J* = 9.0, 3.0 Hz, 2H), 8.02 (s, 1H), 7.73(dd, *J* = 9.0, 3.0 Hz, 1H), 7.01–6.98 (m, 2H), 4.23–4.19 (m, 1H), 4.16–3.99 (m, 2H), 3.84–3.66 (m, 2H), 2.45 (s, 3H), 2.08–1.99 (m, 1H), 1.94–1.77 (m, 2H), 1.72–1.63 (m, 1H); ^13^C NMR (75 MHz, DMSO-*d*_*6*_) *δ* 161.02, 160.64, 152.17, 138.36, 136.91, 132.74, 129.77, 124.92, 122.46, 119.59, 113.10, 111.30, 102.45, 76.80, 71.11, 67.98, 28.06, 25.67, 21.15; HRMS (ESI) m/z calcd for C_19_H_19_O_4_^+^[M + H]^+^: 311.1205 found: 311.1201.

3-((1,3-dioxolan-2-yl) methoxy)-8-methyl-6H-benzo[c]chromen-6-one (**2h**): Using 2-(bromomethyl)-1,3-dioxolane as the starting martial, the desired product **2h** was obtained as a white solid (1.36 g, 60.7%). M. p. 148.2–148.8 °C; IR characteristic peaks appear at (ν cm^−1^): 1722 (–C=O), 1178 (C–O–C), 1275, 1045 (Ar–O–R). ^1^H NMR (300 MHz, DMSO-*d*_*6*_) *δ* 8.22 (d, *J* = 3.0 Hz, 1H), 8.18 (d, *J* = 3.0 Hz, 1H), 8.01 (*s*, 1H), 7.73 (dd, *J* = 9.0, 3.0 Hz, 1H), 7.03–6.99 (m, 2H), 5.24 (t, *J* = 3.0 Hz, 2H), 4.12 (d, *J* = 3.0 Hz, 2H), 4.01–3.85 (m, 4H), 2.45 (s, 3H). ^13^C NMR (75 MHz, DMSO-*d*_6_) *δ* 160.99, 160.22, 152.13, 138.48, 136.93, 132.67, 129.79, 124.98, 122.53, 119.65, 113.08, 111.60, 102.65, 101.56, 69.02, 65.04, 21.16; HRMS (ESI) *m/z* calcd for C_18_H_17_O_5_^+^[M + H]^+^: 312.0998 found: 312.0995.

3-(2-(1,3-dioxolan-2-yl) ethoxy)-8-methyl-6*H*-benzo[c]chromen-6-one (**2i**): Using 2-(2-bromoethyl)- -1,3-dioxolane as the starting martial, the desired product **2i** was obtained as a white solid (1.39 g, 48.4%). M. p. 138.0–139.1 °C; IR characteristic peaks appear at (ν cm^−1^): 1731 (–C=O), 1169 (C–O–C), 1274, 1046 (Ar–O–R). ^1^H NMR (300 MHz, DMSO-*d*_*6*_) *δ* 8.21 (dd, *J* = 6.0, 3.0 Hz, 2H), 8.02 (s, 1H), 7.74 (dd, *J* = 9.0, 3.0 Hz,1H), 7.00–6.97 (m, 2H), 5.02 (t, *J* = 6.0 Hz, 1H), 4.19 (t, *J* = 7.5 Hz, 2H), 3.96–3.92 (m, 2H), 3.83–3.79 (m, 2H), 2.45 (s, 3H), 2.07 (dd, *J* = 12.0, 6.0 Hz, 2H); ^13^C NMR (75 MHz, DMSO-*d*_*6*_) *δ* 161.00, 160.43, 152.19, 138.36, 136.89, 132.71, 129.76, 124.95, 122.45, 119.59, 112.98, 111.33, 102.41, 101.58, 64.78, 64.64, 33.65, 21.15; HRMS (ESI) *m/z* calcd for C_19_H_19_O_5_^+^[M + H]^+^: 326.1154 found: 326.1150.

3-(cyclohexylmethoxy)-8-methyl-6*H*-benzo[c]chromen-6-one (**2j**): Using (bromomethyl)cyclohexane as the starting martial, the desired product **2j** was obtained as a white solid (1.38 g, 48.6%). M. p. 113.3–115.1 °C; IR characteristic peaks appear at (ν cm^−1^): 1726 (–C=O), 1172 (C–O–C), 1274, 1022 (Ar–O–R). ^1^H NMR (300 MHz, DMSO-*d*_*6*_) *δ* 8.21 (dd,* J* = 9.0, 3.0 Hz, 2H), 8.01 (s, 1H), 7.73(dd, *J* = 9.0, 1.9 Hz, 1H), 7.00–6.96 (m, 2H), 389 (d, *J* = 6.0 Hz, 2H), 2.45 (s, 3H), 1.84–1.65(m, 6H), 1.33–1.00 (m, 5H); ^13^C NMR (75 MHz, DMSO-*d*_*6*_) *δ* 161.05, 160.91, 152.23, 138.31, 136.93, 132.81, 129.78, 124.92, 122.45, 119.56, 113.10, 111.11, 102.39, 73.70, 37.42, 29.64, 26.49, 25.70, 21.1; HRMS (ESI) *m/z* calcd for C_21_H_23_O_3_^+^[M + H]^+^: 322.1569 found: 322.1565.

8-methyl-3-((tetrahydro-2*H*-pyran-4-yl) methoxy)-6*H*-benzo[c]chromen-6-one (**2k**): Using 4-(bromomethyl) tetrahydro-2H-pyran as the starting martial, the desired product **2k** was obtained as a white solid (1.32 g, 46.2%). M. p. 148.0–148.6 °C; IR characteristic peaks appear at (ν cm^−1^): 1735 (–C=O), 1180 (C–O–C), 1278, 1034 (Ar–O–R). ^1^H NMR (300 MHz, DMSO-*d*_*6*_) *δ* 8.16 (s, 1H), 7.90 (dd, *J* = 9.0, 2.9 Hz, 2H), 7.60 (dd, *J* = 9.0, 3.0 Hz,1H), 6.89 (dd, *J* = 9.0, 3.0 Hz, 1H), 6.84 (d, *J* = 3.0 Hz,1H), 4.04 (dd, (dd, *J* = 9.0, 3.0 Hz, 2H), 3.87 (d, *J* = 3.0 Hz, 2H), 3.50–3.42 (m, 2H), 2.48 (s, 3H), 2.16–2.05 (m, 1H), 1.81–1.76 (m, 2H), 1.56–1.43(m, 2H); ^13^C NMR (75 MHz, DMSO-*d*_*6*_) *δ* 161.03, 160.74, 15,222, 138.35, 136.92, 132.76, 129.78, 124.94, 122.46, 119.57, 113.09, 111.22, 102.43, 72.99, 67.06, 29.61, 21.25; HRMS (ESI) *m/z* calcd for C_20_H_21_O_4_^+^[M + H]^+^: 325.1362 found: 325.1358.

3-((2-fluorobenzyl) oxy)-8-methyl-6*H*-benzo[c]chromen-6-one(**2l**): Using 1-(bromomethyl)-2- -fluorobenzene as the starting martial, the desired product **2l** was obtained as a white solid (0.92 g, 31.1%). M. p. 154.9–155.5 °C; IR characteristic peaks appear at (ν cm^−1^): 1719 (–C=O), 1173 (C–O–C), 1276, 1043 (Ar–O–R). ^1^H NMR (300 MHz, DMSO-*d*_*6*_) *δ* 8.25 (dd, *J* = 9.0, 3.0 Hz, 2H), 8.03 (s, 1H), 7.75 (d, *J* = 6.0 Hz, 1H), 7.63–7.60 (m, 1H), 7.49–7.44 (m, 1H), 7.28 (dd, *J* = 12.0, 6.0 Hz, 2H), 7.15 (d, *J* = 2.5 Hz, 1H), 7.08 (dd, *J* = 6.0, 3.0 Hz, 1H), 5.27 (s, 2H), 2.46 (s, 3H); ^13^C NMR (75 MHz, DMSO-*d*_*6*_) *δ* 160.99, 160.17, 152.17, 138.50, 136.93, 132.67, 131.47, 131.42, 131.23, 129.80, 125.05, 123.83, 122.54, 119.67, 116.10, 115.82, 113.22, 111.67, 102.83, 64.60, 21.16; HRMS (ESI) *m/z* calcd for C_21_H_16_FO_3_^+^[M + H]^+^: 335.1005 found: 335.1001.

3-(2-(1,3-dioxan-2-yl) ethoxy)-8-methyl-6*H*-benzo[c]chromen-6-one (**2m**): Using 2-(2-bromoethyl)-1,3- -dioxane as the starting martial, the desired product **2m** was obtained as a white solid (1.50 g, 48.0%). M. p. 170.1–172.0 °C; IR characteristic peaks appear at (ν cm^−1^): 1722 (–C=O), 1172 (C–O–C), 1270, 1047 (Ar–O–R). ^1^H NMR (300 MHz, CDCl_3_) *δ* 8.16 (s, 1H), 7.91 (d, *J* = 3.0 Hz, 1H), 7.88 (d, *J* = 3.0 Hz, 1H), 7.61–7.58 (m, 1H), 6.92–6.87 (m, 2H), 4.80 (t, *J* = 6.0, 1H), 4.17–4.10 (m, 4H), 3.80 (td, *J* = 12.0, 3.0 Hz, 2H), 2.48 (s, 3H), 2.12 (q, *J* = 6.0 Hz, 2H), 1.40–1.25 (m, 2H); ^13^C NMR (75 MHz, DMSO-*d*_*6*_) *δ* 161.01, 160.48, 152.20, 138.37, 136.91, 132.73, 129.78, 124.97, 122.47, 119.60, 112.97, 111.32, 102.46, 99.12, 66.57, 64.29, 34.92, 25.84, 21.16; HRMS (ESI) *m/z* calcd for C_20_H_21_O_5_^+^[M + H]^+^: 341.1311 found: 341.1308.

3-((3,5-dimethoxybenzyl) oxy)-8-methyl-6*H*-benzo[c]chromen-6-one (**2n**): Using 1-(bromomethyl)-3,5-dimethoxybenzene as the starting martial, the desired product **2n** was obtained as a white solid (1.18 g, 34.0%). M. p. 158.7–160.2 °C; IR characteristic peaks appear at (ν cm^−1^): 1725 (–C=O), 1172 (C–O–C), 1275, 1038 (Ar–O–R). ^1^H NMR (300 MHz, DMSO-*d*_*6*_) *δ* 8.25 (dd, *J* = 9.0, 3.0 Hz, 2H), 8.02 (s, 1H), 7.75 (d, *J* = 9.0 Hz, 1H), 7.07–7.05 (m, 2H), 6.64 (d, *J* = 3.0 Hz, 2H), 6.47 (s, 1H), 5.17 (s, 2H), 3.75 (s, 6H), 2.45 (s, 3H); ^13^C NMR (75 MHz, DMSO-*d*_*6*_) *δ* 161.69, 161.12, 160.08, 152.17, 138.51, 138.02, 136.14, 132.59, 130.29, 123.55, 121.10, 119.84, 112.99, 111.61, 105.21, 102.74, 100.05, 70.34, 55.41, 29.72, 21.22; HRMS (ESI) *m/z* calcd for C_23_H_21_O_5_^+^[M + H]^+^: 377.1311 found: 377.1308.

#### Synthesis of 3-hydroxy-7,8,9, 10-tetrahydro-6*H*-benzo[C]chromen-6-one (3)

Resorcinol (20 g, 181.6 mmol) and zirconium chloride (4.23 g, 272.4 mmol) were added into a reaction flask, 46.4 mL ethyl 2-oxocyclohexanecarboxylate was slowly added dropwise, and the flask was heated to 70 ℃. The reaction was monitored by TLC and ceased after the raw materials were consumed. The reaction solution was then poured into ice water, filtered under suction, and the filter cake was washed with water until the filtrate was clear and transparent and a crude yellow solid was obtained. The crude product was added to a flask and sufficient ethanol was added to reflux for recrystallisation. This reaction was cut off when the solution was clear and transparent. The liquid was then cooled and a large amount of solid precipitated out. The precipitate was filtered and the pure product of 3-hydroxy-7,8,9,10-tetrahydro-6H-benzo[c]chromen-6-one was obtained (26.91 g, 68.5%). M. p. 199.6–201.3 °C; ^1^H NMR (300 MHz, DMSO-*d*_*6*_) *δ* 10.36 (s, 1H), 7.52 (d, *J* = 9.0 Hz, 1H), 6.77 (dd, *J* = 9.0, 3.0 Hz, 1H), 6.68 (d, *J* = 2.4 Hz, 1H), 2.71 (d, *J* = 3.0 Hz, 2H), 2.38 (d, *J* = 6.0 Hz, 2H), 1.8–1.65 (m, 4H). ^13^C NMR (75 MHz, DMSO-*d*_*6*_) *δ* 161.47, 160.32, 153.50, 148.16, 125.49, 118.89, 113.13, 112.43, 102.38, 25.06, 23.94, 21.72, 21.34.

#### Synthesis of 3-hydroxy-7,8,9,10-tetrahydro-6H-benzo[c]chromen-6-one derivatives (4a–4k)

3-Hydroxy-7,8,9,10-tetrahydro-6*H*-benzo[c]chromen-6-one (3) (2.0 g, 9.2 mmol), anhydrous potassium carbonate (1.7 g, 12 mmol), anhydrous DMF (100 mL), and bromide (12 mmol) were added to a reaction flask, stirred, and heated to 128 °C to reflux. The reaction was monitored by TLC. Once the reaction was complete the mixture was poured into ice water, filtered with suction, and the filter cake was washed with water until the filtrate was clear and transparent. The filter cake was then separated by column chromatography (PE:EA = 30:1) to obtain pure compounds .

3-ethoxy-7,8,9,10-tetrahydro-6*H*-benzo[c]chromen-6-one (4**a**): Using bromoethane as the starting martial, the desired product **4a** was obtained as a white solid (1.32 g, 58.6%). M. p. 104.2–105.6 °C; IR characteristic peaks appear at (ν cm^−1^): 1705 (–C=O), 1166 (C–O–C), 1292, 1041 (Ar–O–R).^1^H NMR (300 MHz, DMSO-*d*_*6*_) *δ* 7.63–7.60 (m, 1H), 6.93–6.90 (m, 2H), 4.11 (q, *J* = 6.0 Hz, 2H), 2.75 (d,* J* = 6.4 Hz, 2H), 2.42–2.38 (m, 2H), 1.74 (td, *J* = 6.3, 3.0 Hz, 4H), 1.35 (t, *J* = 6.0 Hz, 3H). ^13^C NMR (75 MHz, DMSO-*d*_*6*_) *δ* 161.38, 160.94, 153.44, 148.04, 125.38, 119.78, 113.44, 112.64, 101.31, 64.27, 25.09, 23.99, 21.67, 21.31, 14.91; HRMS (ESI) *m/z* calcd for C_15_H_17_O_3_^+^[M + H]^+^: 245.1099 found: 245.1096.

3-propoxy-7,8,9,10-tetrahydro-6*H*-benzo[c]chromen-6-one (**4b**): Using 1-bromopropane as the starting martial, the desired product **4b** was obtained as a white solid (1.24 g, 52.3%). M. p. 107.1–108.3 °C; IR characteristic peaks appear at (ν cm^−1^): 1701 (–C=O), 1171 (C–O–C), 1284, 1040 (Ar–O–R). ^1^H NMR (300 MHz, DMSO-*d*_*6*_) *δ* 7.63–7.60 (m, 1H), 6.95–6.91 (m, 2H), 4.02 (t, *J* = 6.0 Hz, 2H), 2.76 (d, *J* = 6.0 Hz, 2H), 2.41 (d, *J* = 6.0 Hz, 2H), 1.81–1.69 (m, 6H), 0.99 (t, *J* = 7.4 Hz, 3H). ^13^C NMR (75 MHz, DMSO-*d*_*6*_) *δ* 161.36, 161.08, 153.42, 147.98, 125.33, 119.75, 113.41, 112.64, 101.32, 70.01, 25.07, 23.98, 22.34, 21.66, 21.30, 10.79; HRMS (ESI) *m/z* calcd for C_16_H_19_O_3_^+^[M + H]^+^: 259.1256 found: 259.1252.

3-isopropoxy-7,8,9,10-tetrahydro-6*H*-benzo[c]chromen-6-one (**4c**): Using 2-bromopropane as the starting martial, the desired product **4c** was obtained as a white solid (0.77 g, 32.5%). M. p. 85.4–87.5 °C; IR characteristic peaks appear at (ν cm^−1^): 1701 (–C=O), 1154 (C–O–C), 1292, 1033 (Ar–O–R). ^1^H NMR (300 MHz, DMSO-*d*_*6*_) *δ* 7.61 (d, *J* = 6.0 Hz, 1H), 6.98–6.87 (m, 2H), 4.79–4.73 (m, 1H), 2.76 (d, *J* = 6.0 Hz, 2H), 2.41 (*J* = 6.0 Hz, 2H), 1.77–1.71 (m, 4H), 1.30 (d, *J* = 6.0 Hz, 6H). ^13^C NMR (75 MHz, DMSO-*d*_*6*_) *δ* 161.38, 159.91, 153.49, 147.99, 125.43, 119.71, 113.39, 113.32, 102.28, 70.41, 25.06, 23.98, 22.10, 21.68, 21.32; HRMS (ESI) *m/z* calcd for C_16_H_19_O_3_^+^[M + H]^+^: 259.1256 found: 259.1252.

3-(sec-butoxy)-7,8,9,10-tetrahydro-6H-benzo[c]chromen-6-one (**4d**): Using 2-bromobutane as the starting martial, the desired product **4d** was obtained as a white solid (1.54 g, 68.6%). M. p. 74.0–74.2 °C; IR characteristic peaks appear at (ν cm^−1^): 1709 (–C=O), 1150 (C–O–C), 1290, 1030 (Ar–O–R). ^1^H NMR (300 MHz, DMSO-*d*_*6*_) *δ* 7.59 (d, *J* = 9.0 Hz, 1H), 6.92 (s, 1H), 6.89 (d, *J* = 3.0 Hz, 1H), 4.58–4.48 (m, 1H), 2.75 (t,* J* = 6.0 Hz, 2H), 2.39 (t, *J* = 6.0 Hz, 2H), 1.77–1.53 (m, 6H), 1.25 (d,* J* = 6.0 Hz, 3H), 0.93 (t,* J* = 7.5 Hz, 3H). ^13^C NMR (75 MHz, DMSO-*d*_*6*_) *δ* 161.39, 160.26, 153.50, 148.00, 125.45, 119.69, 113.42, 113.33, 102.29, 75.23, 28.86, 25.06, 23.98, 21.68, 21.32, 19.33, 9.90; HRMS (ESI) *m/z* calcd for C_17_H_21_O_3_^+^[M + H]^+^: 272.1412 found: 272.1409.

3-(pentyloxy)-7,8,9,10-tetrahydro-6*H*-benzo[c]chromen-6-one (**4e**): Using 1-bromopentane as the starting martial, the desired product **4e** was obtained as a white solid (0.87 g, 32.9%). M. p. 110.6–112.4 °C; IR characteristic peaks appear at (ν cm^−1^): 1705 (–C=O), 1165 (C–O–C), 1292, 1035 (Ar–O–R). ^1^H NMR (300 MHz, DMSO-*d*_*6*_) *δ* 7.62 (d, *J* = 6.0 Hz, 1H), 6.95–6.92 (m, 2H), 4.06 (t, *J* = 6.0 Hz, 2H), 2.76 (d, *J* = 3.0 Hz, 2H), 2.40 (t, *J* = 6.0 Hz, 2H), 1.77–1.70 (m, 6H), 1.44–1.31 (m, 4H), 0.90 (t, *J* = 4.5 Hz, 3H). ^13^C NMR (75 MHz, DMSO-*d*_*6*_) *δ* 161.39, 161.10, 153.44, 148.03, 125.37, 119.77, 113.43, 112.69, 101.35, 68.57, 28.65, 28.11, 25.09, 23.99, 22.34, 21.67, 21.32, 14.37 ; HRMS (ESI) *m/z* calcd for C_18_H_23_O_3_^+^[M + H]^+^: 287.2569 found: 287.2565.

3-((tetrahydrofuran-2-yl)methoxy)-7,8,9,10-tetrahydro-6*H*-benzo[c]chromen-6-one (**4f**): Using 2-(bromomethyl) tetrahydrofuran as the starting martial, the desired product **4f** was obtained as a white solid (1.26 g, 45.5%). M. p. 112.2–114.0 °C; IR characteristic peaks appear at (ν cm^−1^): 1700 (–C=O), 1169 (C–O–C), 1281, 1037 (Ar–O–R). ^1^H NMR (400 MHz, DMSO-*d*_*6*_) *δ* 7.62 (d, *J* = 8.5 Hz, 1H), 6.95–6.93 (m, 2H), 4.21–4.15 (m, 1H), 4.08 (dd, *J* = 8.0, 4.0 Hz, 1H), 4.01 (dd, *J* = 12.0, 8.0 Hz, 1H), 3.79 (dd, *J* = 16.0, 8.0 Hz, 1H), 3.69 (dd, *J* = 12.0, 4.0 Hz, 1H), 2.77 (d, *J* = 4.0 Hz, 2H), 2.40 (t, *J* = 8.0 Hz, 2H), 2.06–1.98 (m, 1H), 1.92–1.80 (m, 2H), 1.78–1.63 (m, 5H). ^13^C NMR (75 MHz, DMSO-*d*_*6*_) *δ* 161.36, 160.97, 153.39, 148.01, 125.38, 119.88, 113.59, 112.71, 101.46, 76.77, 71.10, 67.97, 28.03, 25.67, 25.09, 24.00, 21.67, 21.31; HRMS (ESI) *m/z* calcd for C_18_H_21_O_4_^+^[M + H]^+^: 301.1362 found: 301.1360.

3-((1,3-dioxolan-2-yl) methoxy)-7,8,9,10-tetrahydro-6*H*-benzo[c]chromen-6-one (**4g)**: Using 2-(bromomethyl)-1,3-dioxolane as the starting martial, the desired product **4g** was obtained as a white solid (1.10 g, 39.5%). M. p. 128.1–129.5 °C; IR characteristic peaks appear at (ν cm^−1^): 1705 (–C=O), 1163 (C–O–C), 1295, 1047 (Ar–O–R). ^1^H NMR (300 MHz, DMSO-*d*_*6*_) *δ* 7.62 (d,* J* = 8.5 Hz, 1H), 6.99–6.94 (m, 2H), 5.23 (t, *J* = 4.5 Hz, 1H), 4.11 (d, *J* = 3.0 Hz, 2H), 3.99–3.84 (m, 4H), 2.76 (d, *J* = 6.0 Hz, 2H), 2.41 (d, *J* = 6.3 Hz, 2H), 1.78–1.70 (m, 4H); ^13^C NMR (75 MHz, DMSO-*d*_*6*_) *δ* 161.33, 160.55, 153.32, 147.98, 125.44, 120.08, 113.86, 112.67, 101.66, 101.52, 68.99, 65.04, 25.10, 24.01, 21.65, 21.30; HRMS (ESI) *m/z* calcd for C_17_H_19_O_5_^+^[M + H]^+^: 303.1154 found: 303.1151.

3-(2-(1,3-dioxolan-2-yl) ethoxy)-7,8,9,10-tetrahydro-6*H*-benzo[c]chromen-6-one (**4h**): Using 2-(2-bromoethyl)-1,3-dioxolane as the starting martial, the desired product **4h** was obtained as a white solid (1.23 g, 42.3%). M. p. 111.0–112.3 °C; IR characteristic peaks appear at (ν cm^−1^): 1701 (–C=O), 1169 (C–O–C), 1281, 1035 (Ar–O–R). ^1^H NMR (400 MHz, DMSO-*d*_*6*_) *δ* 7.62 (d, *J* = 8.0 Hz, 1H), 6.96–6.93 (m, 2H), 5.01 (t, *J* = 4.0 Hz, 1H), 4.17 (t, *J* = 6.0 Hz, 2H), 3.95–3.89 (m, 2H), 3.85–3.79 (m, 2H), 2.76 (d, *J* = 4.0 Hz, 2H), 2.40 (t, *J* = 6.0 Hz, 2H), 2.08–20.4 (m, 2H), 1.78–1.71 (m, 4H). ^13^C NMR (75 MHz, DMSO-*d*_*6*_) *δ* 161.35, 160.77, 153.41, 147.99, 125.43, 119.93, 113.63, 112.60, 101.56, 101.44, 64.77, 64.65, 33.61, 25.09, 24.00, 21.66, 21.30; HRMS (ESI) *m/z* calcd for C_18_H_21_O_5_^+^[M + H]^+^: 317.1131 found: 317.1128.

3-(cyclohexylmethoxy)-7,8,9,10-tetrahydro-6*H*-benzo[c]chromen-6-one (**4i**): Using (bromomethyl) cyclohexane as the starting martial, the desired product **4i** was obtained as a white solid (1.24 g, 43.2%). M. p. 107.2–109.3 °C; IR characteristic peaks appear at (ν cm^−1^): 1707 (–C=O), 1158 (C–O–C), 1295, 1035 (Ar–O–R). ^1^H NMR (300 MHz, DMSO-*d*_*6*_) *δ* 7.62 (d, *J* = 12.0 Hz, 1H), 6.95–6.91 (m, 2H), 3.88 (d, *J* = 6.0 Hz, 2H), 2.76 (d, *J* = 6.0 Hz, 2H), 2.41 (d, *J* = 6.0 Hz, 2H), 1.83–1.65 (m, 10H), 1.32–0.99 (m, 5H). ^13^C NMR (75 MHz, DMSO-*d*_*6*_) *δ* 161.24, 153.45, 148.06, 125.39, 119.77, 113.44, 112.71, 101.42, 73.69, 37.40, 29.62, 26.47, 25.69, 25.10, 24.00, 21.68, 21.32; HRMS (ESI) *m/z* calcd for C_20_H_25_O_3_^+^[M + H]^+^: 313.1725 found: 313.1721.

3-((tetrahydro-2H-pyran-4-yl) methoxy)-7,8,9,10-tetrahydro-6H-benzo[c]chromen-6-one (4j): Using 4-(bromomethyl)tetrahydro-2H-pyran as the starting martial, the desired product **4j** was obtained as a white solid (0.94 g, 41.8%). M. p. 124.6–125.2 °C; IR characteristic peaks appear at (ν cm^−1^): 1701 (–C=O), 1170 (C–O–C), 1280, 1039 (Ar–O–R). ^1^H NMR (300 MHz, DMSO-*d*_*6*_) *δ* 7.61–7.58 (m, 1H), 6.94–6.91 (m, 2H), 3.94–3.86 (m, 4H), 3.29 (d, *J* = 3.0 Hz, 2H), 2.75 (t,* J* = 6.0 Hz, 2H), 2.39 (t, *J* = 6.0 Hz, 2H), 2.08–1.96 (m, 1H), 1.77–1.65 (m, 6H), 1.40–1.20 (m, 2H). ^13^C NMR (75 MHz, DMSO-*d*_*6*_) *δ* 161.36, 161.07, 153.42, 148.00, 125.38, 119.82, 113.52, 112.69, 101.44, 72.98, 67.05, 34.76, 29.59, 25.09, 23.99, 21.66, 21.31; HRMS (ESI) *m/z* calcd for C_19_H_23_O_4_^+^[M + H]^+^: 314.1518 found: 314.1513.

3-(2-(1,3-dioxan-2-yl) ethoxy)-7,8,9,10-tetrahydro-6*H*-benzo[c]chromen-6-one (**4k**) : Using 2-(2-bromoethyl)-1,3-dioxane as the starting martial, the desired product **4k** was obtained as a white solid (1.95 g, 64.2%). M. p. 107.5–108.4 °C; IR characteristic peaks appear at (ν cm^−1^): 1716 (–C=O), 1152 (C–O–C), 1293, 1045 (Ar–O–R). ^1^H NMR (300 MHz, DMSO-*d*_*6*_) *δ* 7.61 (d, *J* = 9.0 Hz, 1H), 6.95–6.91 (m, 2H), 4.74 (t, *J* = 6.0 Hz, 1H), 4.11 (t, *J* = 6.0 Hz, 2H), 4.04–3.99 (m, 2H), 3.72 (td, *J* = 12.0, 3.0 Hz, 2H), 2.75 (d, *J* = 6.0 Hz, 2H), 2.41 (d, *J* = 6.0 Hz, 2H), 1.99–1.73 (m, 7H), 1.35 (d, *J* = 12.0 Hz, 1H); ^13^C NMR (75 MHz, DMSO-*d*_*6*_) *δ* 161.35, 160.81, 153.40, 147.98, 125.42, 119.91, 113.60, 112.57, 101.47, 99.09, 66.56, 64.29, 34.89, 25.83, 25.08, 24.00, 21.66, 21.30; HRMS (ESI) *m/z* calcd for C_19_H_23_O_5_^+^[M + H]^+^: 331.1467 found: 331.1464.

3-((2-fluorobenzyl) oxy)-7,8,9,10-tetrahydro-6*H*-benzo[c]chromen-6-one (**4l**): Using 1-(bromomethyl) -2-fluorobenzene as the starting martial, the desired product **4l** was obtained as a white solid (1.58 g, 52.9%). M. p. 124.2–125.6 °C; IR characteristic peaks appear at (ν cm^−1^): 1711 (–C=O), 1168 (C–O–C), 1278, 1035 (Ar–O–R). ^1^H NMR (400 MHz, DMSO-*d*_*6*_) *δ* 7.66–7.57 (m, 2H), 7.45 (td, *J* = 8.0, 1.8 Hz, 1H), 7.27 (q, *J* = 8.0 Hz, 2H), 7.10 (d,* J* = 2.6 Hz, 1H), 7.02 (dd, *J* = 9.0, 3.0 Hz, 1H), 5.25 (s, 2H), 2.76 (d, *J* = 3.0 Hz, 2H), 2.41 (t, *J* = 6.0 Hz, 2H), 1.78–1.72 (m, 4H). ^13^C NMR (75 MHz, DMSO-*d*_*6*_) *δ* 161.32, 160.48, 153.35, 147.97, 131.43, 131.18, 125.49, 125.06, 123.65, 120.11, 116.09, 115.81, 113.91, 112.82, 101.83, 64.60, 25.09, 24.01, 21.65, 21.29; HRMS (ESI) *m/z* calcd for C_20_H_18_FO_3_^+^[M + H]^+^: 325.1162 found: 325.1159.

3-((3,5-dimethoxybenzyl) oxy)-7,8,9,10-tetrahydro-6*H*-benzo[c]chromen-6-one (**4m**): Using 1-(bromomethyl)-3,5-dimethoxybenzene as the starting martial, the desired product **4m** was obtained as a white solid (2.18 g, 64.6%). M. p. 98.5–99.2 °C; IR characteristic peaks appear at (ν cm^−1^): 1708 (–C=O), 1158 (C–O–C), 1285, 1046 (Ar–O–R). ^1^H NMR (300 MHz, DMSO-*d*_*6*_) *δ* 7.62 (d, *J* = 8.5 Hz, 1H), 7.02–6.99 (m, 2H), 6.62 (d, *J* = 2.3 Hz, 2H), 6.46 (t, *J* = 2.3 Hz, 1H), 5.14 (s, 2H), 3.74 (s, 6H), 2.76 (d, *J* = 3.0 Hz, 2H), 2.40 (t, *J* = 6.0 Hz, 2H), 1.84–1.73 (m, 4H). ^13^C NMR (75 MHz, DMSO-*d*_*6*_) *δ* 161.05, 160.58, 153.33, 148.01, 139.24, 125.47, 120.02, 113.78, 112.97, 106.01, 101.95, 100.03, 70.04, 55.68, 25.10, 24.01, 21.66, 21.30; HRMS (ESI) *m/z* calcd for C_22_H_23_O_5_^+^[M + H]^+^: 367.1467 found: 367.1463.

### PDE2 enzyme inhibitory activity experiment

Phosphodiesterase II (PDE2) was used as the target, BAY60-7550 was employed as a positive control, and the compounds 2a-2n and 4a-4 m were tested for their PDE2 inhibitory activity using the AlphaScreen® cAMP kit method. *E. coli* BL21-Codon Plus (DE3) and DH5α were purchased from Stratagene, and the recombinant plasmid pET15b-PDE2A (580–941) was donated by the Luo Haibin Laboratory of Sun Yat-sen University. For the expression and purification of PDE2 protein and specific methods of AlphaScreen, please refer to something similar to this^33,35^.

## Conclusion

27 derivatives were designed and synthesized using 3-hydroxy-8-methyl-6*H*-benzo[C]chromen-6-one and 3-hydroxy-7,8,9, 10-tetrahydro-6*H*-benzo[C] chromen-6-one as the lead compounds, and the inhibitory activities of each derivative against PDE2 were tested at the enzyme level. The results showed that the **2e, 2j,** and **4c** yield higher inhibitory activity against PDE2 than the lead compounds, with **2e** and **4c** exhibiting the best inhibitory activity against PDE2 with IC_50_ values of 33.95 μM and 34.35 μM, respectively. The inhibitory activity of the 6*H*-benzo[C]chromen-6-one derivatives on PDE2 was found to be better when the substituent was an alkane, and especially when the number of carbons was about five^33^ which strongly agrees with the previous findings. Suggestions are provided from the aspect of a general rule for the combination of 6*H*-benzo[C]chromen-6-one derivatives with the crystal structure 4HTX of the PDE2 protein. This investigation lay the foundation for further structural modification of the lead compounds, and for designing small PDE2 inhibitor molecules with improved inhibitory activity.

## Abbreviations

EA: Ellagic acid
AD: Alzheimer's disease
PD: Parkinson's disease
PDE2: Phosphodiesterase II
cAMP: Cyclic adenosine monophosphate
cGMP: Cyclic guanosine monophosphate
